# Effect of Telephone-Based Support on Postpartum Depression: A Randomized Controlled Trial

**DOI:** 10.22074/ijfs.2015.4246

**Published:** 2015-07-27

**Authors:** Hourieh Shamshiri Milani, Eznollah Azargashb, Narges Beyraghi, Sara Defaie, Taha Asbaghi

**Affiliations:** 1 Infertility and Reproductive Health Research Center (IRHRC), Shahid Beheshti University of Medical Sciences, Tehran, Iran; 2 Department of Health and Social Medicine, School of Medicine, Shahid Beheshti University of Medical Sciences, Tehran, Iran; 3 Taleghani Hospital, Shahid Beheshti University of Medical Sciences, Tehran, Iran

**Keywords:** Postpartum Depression, Postnatal Care, Volunteers, Mother, Women Health

## Abstract

**Background:**

Postpartum depression (PPD) is one public health issue that affects both maternal and child health. This research studies the effect of health volunteers’ telephone- based support on decreasing PPD.

**Materials and Methods:**

This randomized controlled trial evaluated 203 women who had uncomplicated deliveries. The women completed the Edinburg Postnatal Depres- sion Scale (EPDS), 10 to 15 days after childbirth in order to be assessed for pre-trial depression scores. The cut-off point for depression was considered to be a score of >10. We randomly assigned 54 eligible mothers (n=27 per group) with mild and moderate de- pression to the intervention and control groups. In both groups, mothers received routine postpartum care. The intervention group additionally received telephone support from health volunteers. A questionnaire was used to gather demographic and obstetric infor- mation. By the end of the 6thweek, mothers completed the EPDS to be reassessed for depression after intervention. Data were analyzed using the chi-square, Fisher’s exact, t- and paired t tests.

**Results:**

The mean depression scores before intervention (10 to 15 days after childbirth) in the intervention and control groups did not significantly differ (P=0.682). Depres- sion scores of the intervention and control groups showed a significant difference after 6 weeks (P=0.035). In addition, there was a significant decrease in depression for the intervention and control groups (P=0.045).

**Conclusion:**

Health volunteer telephone-based support effectively decreased PPD and may be beneficial to women with symptoms of mild and moderate PPD (Registration number: IRCT201202159027N1).

## Introduction

Pregnancy and childbearing are important events in women’s lives that greatly impact the physical, mental and social health of the mother. Postpartum blues, depression and anxiety are common realities in today’s world, especially in developing countries where many remain underdiagnosed and undertreated due to different reasons (poverty, culture, values and major life events) (1-3). Major depression after childbirth has serious risks such as suicide and infanticide (4, 5). It also decreases breastfeeding, induces severe malnutrition and other diseases (6). Postpartum depression (PPD) is a public health issue whose worldwide incidence is approximately 15% or more (7-11). In some cities of Iran, PPD incidence has been reported as 23.7 and 32% (12, 13).

Finding strategies for prevention, early detection and treatment of PPD can have major benefits in the areas of reproductive rights, as well as medical and financial aspects. These strategies can promote public health, especially in societies with limited human and financial resources. Several strategies have been implemented in an attempt to find helpseeking behaviors, that screen, prevent and manage mothers who suffer from this illness (e.g., peer support); provide telephone care management; and establish consultation centers and Crisis Intervention Units (10, 14-18).

Some studies have shown that women preferred "talking therapies" with someone who was nonjudgmental rather than pharmacological interventions. Dennis and Chung-Lee (19, 20) studied the effect of telephone-based peer support on prevention of PPD at 12 weeks after childbirth. They found that the group who received postpartum care plus telephone-based peer support reported higher levels of positive relationship qualities than the control group. In their study women preferred to talk with someone than to receive medical interventions. They concluded that peer volunteer support might be a preventive strategy for PPD. Simon et al. (17) compared the benefits of telephone care management with telephone psychotherapy for depression and concluded that telephone psychotherapy was more effective than telephone care management.

In Iran, a limited number of studies have described and explained PPD. Hassan Zahraee et al. (21) studied the supportive role of the midwife in preventing PPD in Isfahan. The results showed that mean depression scores of mothers who received emotional and informational support of a midwife on the 2^nd^ and 10^th^ days postpartum were significantly lower than the control group on the 45th day after childbirth. They concluded that midwife support might be an effective factor in preventing PPD. Sadr et al. (22) studied factors that affected PPD in Tehran and have reported significant relationships between PPD and the husband’s education, marital dissatisfaction and lack of social support, unwanted pregnancy, congenital disorders of the newborn, and mental disorders. Additionally, it is possible that genetics may play a role in susceptibility to PPD (2). However, it is important to emphasize that genetic and biologic factors of PPD have yet to be efficiently studied and explained (23).

Telephone-based support can help mothers with access to postpartum information and care as well as receive responses to their questions according to a study by Goodman (24). In Iran, there have not been any studies regarding the effect of health volunteer telephone-based support on PPD. Thus, this research aimed to study the effect of health volunteers’ telephone based-support on PPD. The term "health volunteers" were women who interacted with families in primary health care (PHC) and acted as bridges between health centers and the community in Iran.

## Materials and Methods

In this randomized controlled trial, 203 postpartum women completed the Edinburg Postnatal Depression Scale (EPDS) during routine postpartum care visits conducted 10 to 15 days after uncomplicated childbirth in hospitals affiliated with Shahid Beheshti University of Medical Sciences. The EPDS was validated in Iran. This scale is a 10-item self-report questionnaire with a possible score range of 0 to 30. Items 1, 2, and 4 are scored 0, 1, 2 or 3 with the top box scored as 0 and the bottom box scored as 3. Items 3 and 5-10 are reverse scored, with the top box scored as 3 and the bottom box scored as 0. The cut-off point for detecting depression was identified as a score of >10 (25). Cases with scores ≥14 were considered to be severely depressed. The aim, benefits and privacy were explained in simple words to participants after which the EPDS was completed by the mothers. If the mother was unable to complete the questionnaire, the interviewer assisted with completion. Thus, 75 cases of mothers who suffered from PPD were detected, 67 cases had moderate depression and the other 8 cases had severe depression.

### Inclusion criteria

The criteria for including cases were term pregnancy, live birth and depression score (EPDS score) >10 to <14.

### Exclusion criteria

The criteria to exclude cases were: history of mental disorder, episodes of mental disorders during pregnancy or in the last 12 months that necessitated the use of medicines, nonviable fetus, history of PPD, current use of prescribed psychiatric drugs and an EPDS score of ≥14. Mothers with EPDS scores ≥14 and who had suicidal thoughts were referred to a psychiatrist. By considering the exclusion criteria, 21 of 75 depressed mothers were excluded; 54 of the participants were eligible to remain in the trial.

### Intervention description and assessment

There were 54 eligible mothers out of 203 postpartum mothers (n=27 per group) who had mild to moderate depression (>10 to <14 EPDS scores). These cases were recruited and randomly assigned into the intervention and control groups. After obtaining informed written consent, demographic and obstetric information of the participants was gathered by a questionnaire as follows: age, parity, number of children, job, education, interpersonal relationship with husband, history of disease, newborn gender, willingness of parents to a specific newborn gender, quality of childbirth, type of childbirth, health status of newborn, and wanted pregnancy.

In both groups, mothers received routine postpartum care as shown in table 1. Routine postpartum care did not have any screening tool for PPD. The intervention group received telephone support provided by eight health volunteers. These health volunteers had been trained in a workshop to enable them to communicate effectively and accurately with mothers and to manage their problems. Volunteers were instructed to refer cases that they could not handle to health centers.

Each trained volunteer called 3 to 4 mothers at intervals of 2 to 3 times per week until 6 weeks after childbirth. During each phone call, after a greeting, they asked the mother about her health status, newborn’s condition, complaints, and the mother’s relationship with her newborn or husband, and whether there was any problem. The volunteers managed the problem according to their training, and if needed, assistance was given by a principal researcher. During the conduction of research all volunteers were in contact with the main researcher however they kept all data confidential from their fellow volunteers. The control group did not receive any further intervention during the study. At the end of 6 weeks (after intervention), both groups completed the EPDS to reassess depression scores. There were 5 participants from the intervention group and 3 from the control group that were lost to follow up.

### Data analysis plan

Data were processed using SPSS version 16. Data with nominal and ordinal scales were compared in two groups using the Chi-square and Fisher’s exact tests. Data with ratio scales was tested using the t test. The changes in depression scores between the pre- and post-intervention in each group were tested using the paired t test; the changes of scores between two groups were compared with the t test (26, Table 2). A P value of <0.05 was considered significant.

This study was registered at the Iranian Registry of Clinical Trials (IRCT) website with registration ID: IRCT201202159027N1 and was approved by the Ethics Committee of research at Shahid Behaeshti University of Medical Sciences.

**Table 1:** Routine postpartum care in Iran


Routine postpartum care (until day 42)	Examples

Medical history	Vaginal bleeding and discharge, urinary complaints, psychological status, etc.
Clinical examinations	Vital signs, breasts, abdomen, eyes, etc.
Education and counseling	Nutrition, breastfeeding, family planning, etc.
Nutritional supplements	Ferrous tablets, multivitamins


**Table 2:** Baseline characteristics of study group participants


	Intervention (n=22)	Control (n=24)	P value

Age (Y, Mean ± SD)	27.59 ± 4.81	28.37 ± 7.37	0.67
Parity (Mean ± SD)	2.36 ± 1.22	2.12 ± 1.8	0.596
Number of children (Mean ± SD)	2.90 ± 1.15	2.3 ± 1.4	0.121
Job (%)			
Housewife	19 (86.4%)	21 (87.5%)	0.909
Employed	3 (13.6%)	3 (12.5%)	
Educational level (%)			
<Diploma	13 (61.9%)	15 (68.2%)	0.731
‰¥Diploma	9 (42.8%)	7 (31.8%)	
Relationship with husband (%)			
Good	19 (86.4%)	22 (91.7%)	0.658
Poor	3 (13.6%)	2 (8.3%)	
History of physical disease (%)			
Positive	5 (22.7%)	7 (29.2%)	0.872
Negative	17 (77.3%)	17 (70.8%)	
Newborn gender (%)			
Willingness	12 (54.5%)	15 (62.5%)	0.804
Unwillingness	10 (45.5%)	9 (37.5%)	
Quality of childbirth (%)			
Moderate	8 (36.4%)	7 (29.2%)	0.74
Difficult	10 (45.5%)	13 (54.2%)	
Type of childbirth (%)			
NVD	9 (40.9%)	7 (29.2%)	0.672
Cesarean	13 (59.1%)	16 (66.7%)	
Health status of newborn (%)			
Healthy	14 (63.6%)	18 (75%)	0.606
Unhealthy	8 (36.4%)	6 (25%)	
Wanted pregnancy (%)			
Yes	10 (45.5%)	18 (75%)	0.08
No	12 (54.5%)	6 (25%)	


P<0.05 was considered significant. NVD; Normal vaginal delivery.

## Results

Out of 203 mothers who completed the EPDS, 67 (33%) had mild and moderate depression whereas 8 (4%) had severe depression. The intervention and control groups were the same in baseline characteristics as well as depression scores before intervention. Thus, there were no any significant differences between the intervention and control groups. Contextual variables such as age, parity, number of children, educational level, and relationship with husband were compared in the intervention and control groups as shown in table 2.

Table 3 shows the mean ± SD depression measured by the EPDS scoring scale that included 0, 1, 2 and 3 values as well as changes before and after intervention in the intervention and control groups. Before intervention, the mean ± SD depression score in the intervention group was 12.68 ± 1.35 and in the control group it was 12.83 ± 1.12. There was no significant difference between the groups (P=0.682).

After intervention, the mean depression scores were 7.95 ± 3.45 for the intervention group and 10.33 ± 3.93 for the control group, which significantly differed (P=0.035). Changes in mean depression scores for both the intervention (4.73 ± 3.83-P≤0.001) and control (2.5 ± 3.51-P=0.008) groups significantly differed. A comparison of decrease in depression between the intervention (4.7 scores) and control (2.5 scores) groups showed significantly more decrease in the intervention group than the control group (P=0.045).

**Table 3:** Comparison of mean depression scores and changes in intervention and control groups before and after intervention


	Before	After	Changes	P value
	(Mean ± SD)	(Mean ± SD)	(Mean ± SD)		

Intervention group	12.68 ± 1.35	7.95 ± 3.45	-4.73 ± 3.83	<0.001
C o n t r o l group	12.83 ± 1.12	10.33 ± 3.93	-2.5 ± 3.51	0.008
P value	0.682	0.035	0.045	


P<0.05 was considered significant.

## Discussion

The incidence of PPD in this study was 36.9% whereas in other Iranian studies this incidence was reported as 23.7 (12) and 32% (13). It should be mentioned that the timing of screening differed among these studies therefore these differences could be time dependent. The PPD incidence in developed countries has been reported as 13% (27). Thus the PPD incidence in Iran appeared to be more than developed countries. Further researches should be done for understanding the influencing factors.

Mean depression scores before intervention in both groups were the same, however after intervention the mean depression scores in the intervention group who received telephone support was significantly lower than the control group. It could be concluded that the decrease of EPDS scores was due to intervention. However, we could not find any study about health volunteer telephonebased support. A similar result was reported by Dennis et al. (16) after peer support. They found that PPD could be decreased after peer support (mother to mother support). Meanwhile, Hassan Zahraee et al. (21) found that midwives’ emotional and informational support given on days 2 and 10 after childbirth prevented PPD after 5 weeks. Hantsoo et al. (28) studied the efficacy of treatment with computer-assisted cognitive-behavioral therapy for antepartum depression and suggested this therapy to be used for antepartum depression.

In both groups the depression scores decreased after 6 weeks, but the intervention group showed more decrease than the control group. Mean depression scores of the intervention group decreased in the non-depressed area (decreased under <10 scores), but the control group mean scores remained at the >10 scores (depressed area). In other words, although depression scores in the control group decreased, this was not adequate. A similar result was reported by Dennis et al. (16), they found that in the peer supported group, there were a significantly less proportion of depressed mothers compared to the control group. In contrast, Mohammad-Alizadeh-Charandabi et al. (29) conducted a randomized controlled trial that studied the effect of telephone support on PPD. They found no significant difference regarding frequency of depression as well as mean depression scores between the intervention group that received telephone support by a midwife and the control group. This contradiction might be due to the selection of a different cut-off point for depression. In their trial, the cut-off score was ≥13, whereas in the current study, it was >10, according to findings by Edmondson et al. (25).

The advantage of the findings of the present study when compared with those of other studies and strategies suggested for handling PPD was the decrease in PPD scores which was achieved by engaging inexpert health volunteers and via telephone calls. This was a cost benefit method available in every house. In other words, there was no high-skilled person required, nor the need for any advanced technology or complex tools. In the study by Mohammad-Alizadeh- Charandabi et al. (29), the intervention was performed by trained midwives with no evidence of decreasing PPD reported. In our study, intervention was performed by health volunteers (grassroots community members with a high potential for communication) who worked free of charge and communicated directly with families. This could be used as a model to engage accessible human resources for improving mothers’ health in every society. This model, as a simple method, could reduce the burden of disease and prevent the establishment of depression and its severity. Therefore the use of expensive therapies and medications alongside with complications for families and society would be decreased and the national health system could additionally benefit from this approach. Some studies have suggested computer-assisted cognitive-behavioral therapy for antepartum depression (28). Since the computer is not widely used by Iranian women, this cannot be implemented in Iran at the present time.

The present study limitation included support from the family and the husband as an important factor in PPD, which we have not considered in this study. Thus, additional studies with more cases and factorial designs will be necessary to achieve a precise conclusion.

## Conclusion

In this study, health volunteer telephone-based support was effective in decreasing PPD and may be beneficial to women with mild and moderate levels of PPD symptoms.
